# Concrete Infill Monitoring in Concrete-Filled FRP Tubes Using a PZT-Based Ultrasonic Time-of-Flight Method

**DOI:** 10.3390/s16122083

**Published:** 2016-12-07

**Authors:** Mingzhang Luo, Weijie Li, Chuang Hei, Gangbing Song

**Affiliations:** 1 Electronics and Information School, Yangtze University, Jingzhou 434023, China; luomingzh@163.com (M.L.); heichuang@126.com (C.H.); 2 Department of Mechanical Engineering, University of Houston, Houston, TX 77004, USA; wli27@uh.edu; 3 School of Civil Engineering, Dalian University of Technology, Dalian 116023, China

**Keywords:** concrete-filled FRP tubes, concrete infill condition, lead zirconate titanate (PZT), ultrasonic time of flight

## Abstract

Concrete-filled fiber-reinforced polymer tubes (CFFTs) have attracted interest for their structural applications in corrosive environments. However, a weak interfacial strength between the fiber-reinforced polymer (FRP) tube and the concrete infill may develop due to concrete shrinkage and inadequate concrete compaction during concrete casting, which will destroy the confinement effect and thereby reduce the load bearing capacity of a CFFT. In this paper, the lead zirconate titanate (PZT)-based ultrasonic time-of-flight (TOF) method was adopted to assess the concrete infill condition of CFFTs. The basic idea of this method is that the velocity of the ultrasonic wave propagation in the FRP material is about half of that in concrete material. Any voids or debonding created along the interface between the FRP tube and the concrete will delay the arrival time between the pairs of PZT transducers. A comparison of the arrival times of the PZT pairs between the intact and the defected CFFT was made to assess the severity of the voids or the debonding. The feasibility of the methodology was analyzed using a finite-difference time-domain-based numerical simulation. Experiments were setup to validate the numerical results, which showed good agreement with the numerical findings. The results showed that the ultrasonic time-of-flight method is able to detect the concrete infill condition of CFFTs.

## 1. Introduction

Concrete-filled fiber-reinforced polymer tubes (CFFTs), as a type of hybrid compression member, are attractive for their use as composite materials in several special structural applications including piles, columns, bridge piers, poles, and highway overhead sign structures [1]. Owing to the light weight, high strength, and corrosion resistance of fiber-reinforced polymer (FRP) material, this hybrid provides an excellent alternative to conventional concrete-filled steel tubes (CFSTs) in corrosive situations, particularly the tidal zones of marine piles and the splash zones of highway pavements, which are subjected to deicing salt invasion. A CFFT has two main structural components: a FRP tube and a plain or steel-reinforced concrete infill. When this hybrid item is under compression, the concrete core is simultaneously subjected to axial compression and lateral confinement from the FRP tube, which is in a tensile stress state in the circumferential direction. This lateral confinement from the FRP tube can greatly enhance both the loading strength and the ductility of the concrete [2]. To achieve the improved composite strength, with the loading being transferred between these two components effectively, it is necessary to ensure excellent bonding between the FRP tube and the infilled concrete. However, a weak interface between the FRP tube and the concrete may develop due to concrete shrinkage and inadequate concrete compaction during the concrete casting, which will impair the confinement effects and reduce the load bearing capacity of the CFFT. Therefore, effective detection measures based on nondestructive testing (NDT) methods should be taken to assess the concrete infill condition of the CFFT. The concrete infill condition investigated in this paper refers to the completeness of the infilled concrete inside the FRP tube.

Several NDT methods, such as the ultrasonic pulse velocity method [3,4], acoustic emission [5,6,7,8], infrared thermography [9,10] and the pulse-echo method [11], have been applied in structural damage detection. These methods have shown various degrees of success in assessing structural damage severity. However, these methods require substantial access to the structures under test, which is not always the case, especially for large-scale structures and underground structures. Recently, piezoelectric materials have been emerging as a promising structural diagnostic tool due to their commercial availability, low-cost, and wide bandwidth [12,13]. The most widely used piezoelectric material is lead zirconate titanate (PZT), which can be either mounted on the surface of existing structures or embedded in new structures [14]. Piezoelectric transducer-based methods are becoming popular in interfacial debonding detection for various structures, including steel reinforced concrete [15], concrete-filled steel tubes [16], and concrete-encased composite structures [17,18]. It was determined from a literature survey, however, that there is a very limited volume of research on the concrete infill detection of concrete-filled composite tubes. Very recently, Dong et al. [19] investigated the feasibility of using ultrasound travel time to quantify voids in concrete-filled steel tubes. Similar to Dong’s methodology, this study investigated the efficacy of an ultrasonic time-of-flight (TOF) method to determine the concrete infill condition of CFFTs. In addition, active sensing methods using PZT transducers have been researched in damage detection of various structures [20,21,22,23].

In this study, PZT patches were adopted as transmitters and receivers of ultrasonic waves to monitor the concrete infill for concrete-filled fiber-reinforced polymer tubes by using the ultrasonic TOF. The PZT patches were bonded on the outer surface of the CFFT. The existence of voids inside the CFFT alters the ultrasonic wave propagation path between the PZT transmitter and the PZT receiver. The proposed method is developed to monitor the concrete infill condition for concrete-filled FRP tubes. The basic principle of ultrasonic TOF to detect the defects in CFFTs is that the velocity of the ultrasonic wave propagation in FRP material is about half of that in concrete material. Any voids along the interface between the concrete and the wall of the FRP tube will drive the ultrasonic wave travel along the wall of the FRP tube. By investigating the arrival time of the equally spaced PZT receivers on the FRP tube, the concrete infill condition and the location of the defects can be identified. The methodology was first analyzed using a numerical simulation approach based on the finite-difference time-domain method. The numerical analysis was then validated through the experimental investigations, which involved four different infill conditions—an empty concrete infill, a 1/3 concrete infill, a 2/3 concrete infill, and a 100% or complete concrete infill. The proposed method can be adopted to new structures for concrete infill monitoring during concrete casting and to existing structures for void detection and determination.

## 2. Numerical Simulation Based on the Finite-Difference Time-Domain Method

**Figure 1:**
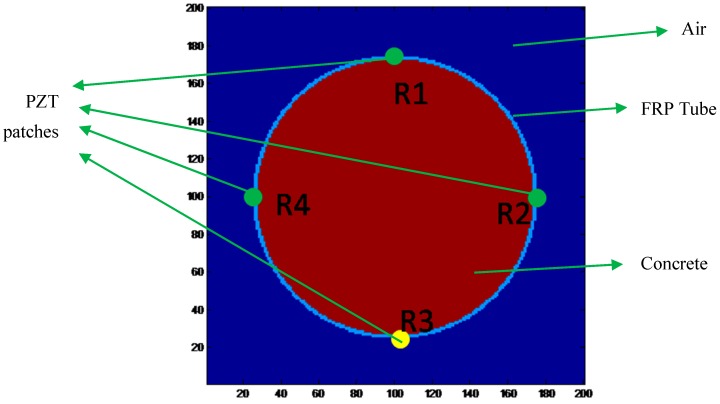
Geometry of the numerical model of the concrete-filled fiber-reinforced polymer tube (CFFT).

The CFFT under analysis consisted of an FRP tube and a concrete infill. The filling condition of the concrete directly affects the load bearing strength of the CFFT. Therefore, different filling conditions of the concrete inside the FRP tube were simulated numerically, and the wave traveling characteristics were analyzed. The geometry of the numerical model is shown in Figure 1. The concrete infill was encased by a FRP tube, and four PZT transducers were modeled. The PZT transducers can be used as either transmitters or receivers, which were equally arranged 90 degrees apart along the circumferential direction of the tube. A layer of ambient air was also established. The properties of the model are shown in Table 1. Note that the ultrasonic wave velocities in FRP material are about half of those in concrete material. The conditions of the concrete infill can be determined by comparing the arrival time of the waves.

The finite-difference time-domain (FDTD) method was adopted to simulate propagation characteristics of the ultrasonic waves in the CFFT model. In order to improve the computing efficiency and reduce the wave distortion, the staggered-grid technique was used, and a perfectly matched layer (PML)-absorbing boundary condition was also applied during the numerical simulation. The Richer wavelet with a frequency of 20 kHz was chosen as the excitation wave. The time step (Δt) and spatial step (Δx, Δy) satisfy $\Delta t=1/\left(v_{\max}\sqrt{2\left(\Delta x^{-2}+\Delta y^{-2}\right)}\right)$, where the $v_{\max}$ is the maximum propagation velocity of the wave.

**Figure 2:**
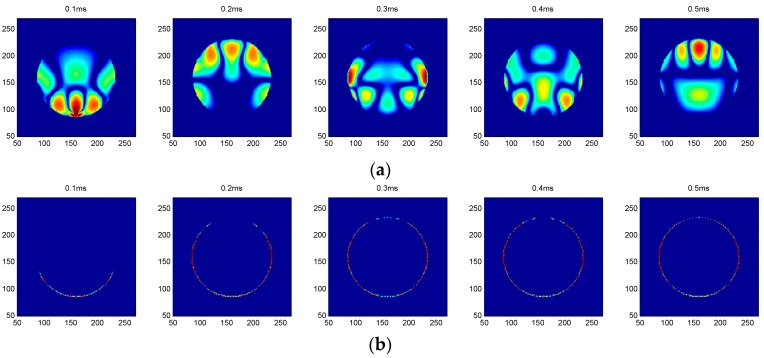
Snapshots excited by R3: (**a**) a full concrete infill and (**b**) an empty concrete infill.

Figure 2 illustrates the snapshots excited by the R3 transmitter over time for both empty concrete and full concrete infills. As can be seen from Figure 2a, when the FRP tube was fully filled with concrete, the wave energy was concentrated at the concrete region because the waves propagated faster in concrete. The waves were received by R2 and R4 receivers first and were then received by R1. As for comparison, the snapshots were also analyzed when the FRP tube was free of concrete. Unlike the case of a full concrete infill, where the wave energy concentrated at the concrete, the wave energy was propagated via the wall of the FRP tube.

**Figure 3:**
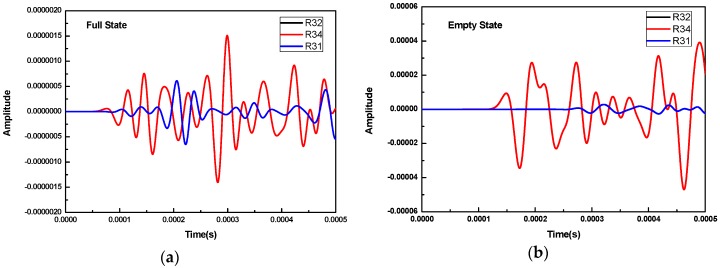
The waveforms received by R1, R2, and R4 when R3 was the transmitter: (**a**) full concrete infill; (**b**) empty concrete infill.

Furthermore, Figure 3 shows the waveforms received by R1, R2, and R4 when R3 was used as the transmitter for both full concrete and empty concrete infills. In the notation of Rij, i represents the number of the transmitter and j represents the number of the receiver. For example, R32 denotes the waveforms received by R2 when R3 is used as the excitation source. As can be seen from these two cases, the wave was picked up by R2 and R4 first and was then received by R1 since R2 and R4 were located closer to the transmitter R3. The wave amplitudes by R2 and R4 are higher. Additionally, the waveforms received by R2 and R4 overlap with each other because they have equal distance to the transmitter R3 and the same wave propagation medium. It should be noted that, for the case of the full concrete infill, the wave travels with the velocity of that in concrete medium; meanwhile, for the case of the empty concrete infill, the wave travels with the velocity of that in the FRP material.

**Figure 4:**
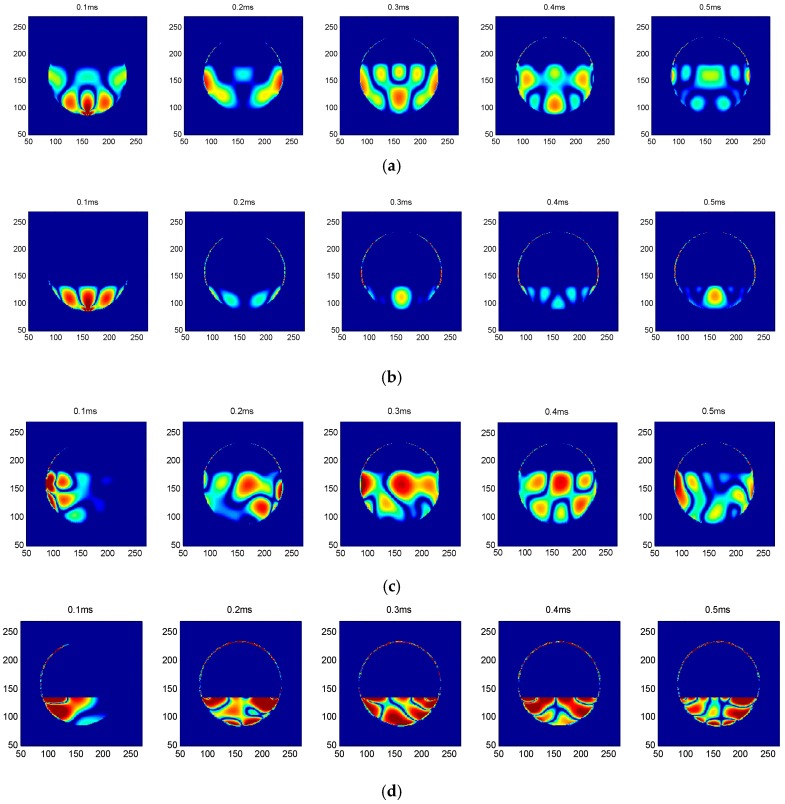
Snapshots of the 1/3 and 2/3 concrete infill conditions excited by R3 or R4: (**a**) the 2/3 concrete infill condition, excited by R3; (**b**) the 1/3 concrete infill condition, excited by R3; (**c**) the 2/3 concrete infill condition, excited by R4; (**d**) the 1/3 concrete infill condition, excited by R4.

As can be seen from the above analysis, the full and empty concrete infill conditions can be easily differentiated by analyzing the ultrasonic time of flight. However, in practical engineering, the concrete is normally partially absent inside the FRP tube due to inadequate compaction, creating voids and debonding defects. To qualitatively determine the concrete infill condition and locate the defects, a cross-excitation method was adopted. Two other different concrete infill conditions, i.e., the 1/3 and 2/3 concrete infill conditions, were examined using R3 and R4 as excitation sources, respectively. Figure 4 shows snapshots of the 1/3 and 2/3 concrete infill conditions excited by R3 and R4, respectively. It can be seen that the snapshots excited by R3 are entirely different from those excited by R4. The snapshots excited by transmitter R3 are symmetrical, while those by R4 are nonsymmetrical. The results from the cross-excitation can be used to determine the occurrence of debonding and obtain the approximate location of the defect.

**Figure 5:**
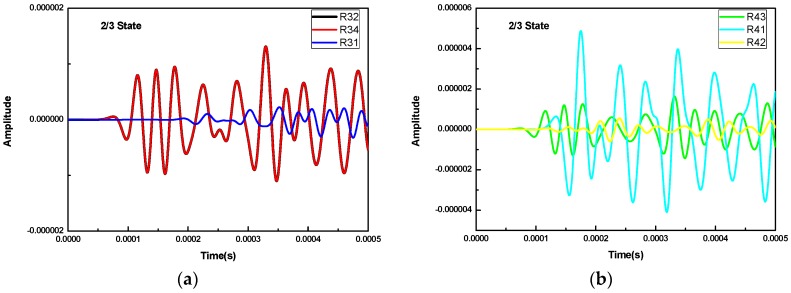

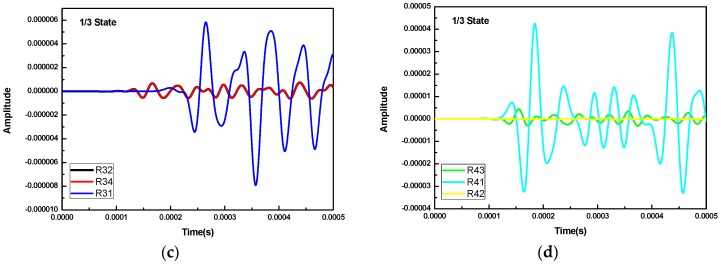
The waveforms of cross-excitation: (**a**) the 2/3 concrete infill condition, excited by R3; (**b**) the 2/3 concrete infill condition, excited by R4; (**c**) the 1/3 concrete infill condition, excited by R3; (**d**) the 1/3 concrete infill condition, excited by R4.

The waveforms of cross-excitation for the 1/3 and 2/3 concrete infill conditions are shown in Figure 5. For the case of the 2/3 concrete infill condition, when R3 is used as excitation source, as shown in Figure 5a, the waveforms received by R2 and R4 have the same arrival time and waveform amplitude, while the one received by R1 is lagging behind in arrival time and has a weaker amplitude. When R4 is used as an excitation source, as shown in Figure 5b, the waveform received by R3 is the same as R34, which share the same arrival time and amplitude. The waveform received by R1 has a large amplitude because the waveform propagates via the wall of the FRP tube. The waveform received by R2, mainly propagating through the concrete material, is the same as R31 in the full concrete infill condition as shown in Figure 3a.

**Figure 6:**
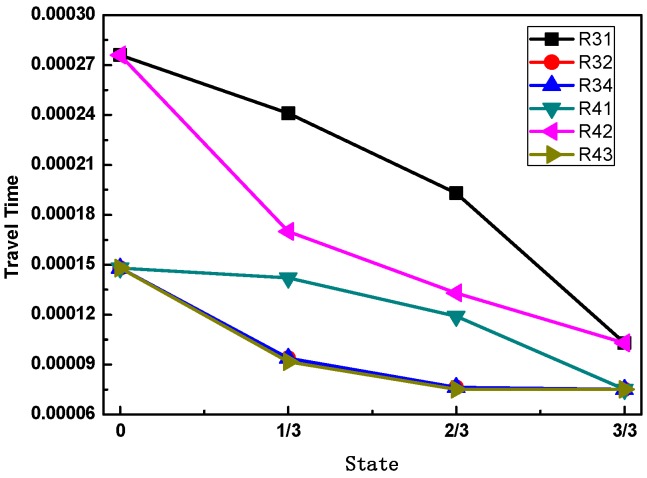
The arrival time of different concrete infill conditions.

For the case of the 1/3 concrete infill condition, when R3 is used as the excitation source, as shown in Figure 5c, the waveforms received by R2 and R4 are the same in terms of arrival time and amplitude. The waveform received by R1 is substantially stronger than the one in the 2/3 concrete infill condition as the wave mainly propagates via the wall of the FRP tube. When R4 is used as the excitation source, as shown in Figure 5d, the waveform received by R3 propagates through both the concrete and the wall of the FRP tube. The waveform received by R1 propagates through the wall of the FRP tube and the one by R2 mainly via the concrete material. From the results of the above numerical simulation, the arrival time and the wave amplitude of the received waveforms for different receivers contain useful information about the concrete infill condition inside the FRP tube. Figure 6 shows the arrival time of different receivers for different concrete infill conditions. As can be seen that for both the empty (0) and the full (3/3) concrete infills, the waveforms of the receivers close to the excitation source, such as R32, R34, R41, and R43, share the same arrival time because they have the same propagation medium and distance. For the same reason, the waveforms of the receivers located farther away from the excitation source, such as R31 and R42, show the same arrival time. For the cases of the 1/3 and 2/3 concrete infills, the arrival time of the receivers shows different characteristics due to the different path and material of the propagation waves. In general, for the full concrete infill condition, the wave travels with the velocity in concrete material, and the time of flight between the transmitter and any receiver is a minimum value. If a void is created in between the transmitter and any receiver, as is the case for the 1/3 and 2/3 concrete infill conditions, the wave propagation path will be directed to the FRP tube before arriving the receiver, thus increasing the ultrasonic TOF. The ultrasonic TOF is at a maximum when there is no concrete infill in the CFFT. Such characteristics can then be utilized to identify the concrete infill condition inside a FRP tube and determine the location of the defects.

## 3. Experimental Verification

**Figure 7:**
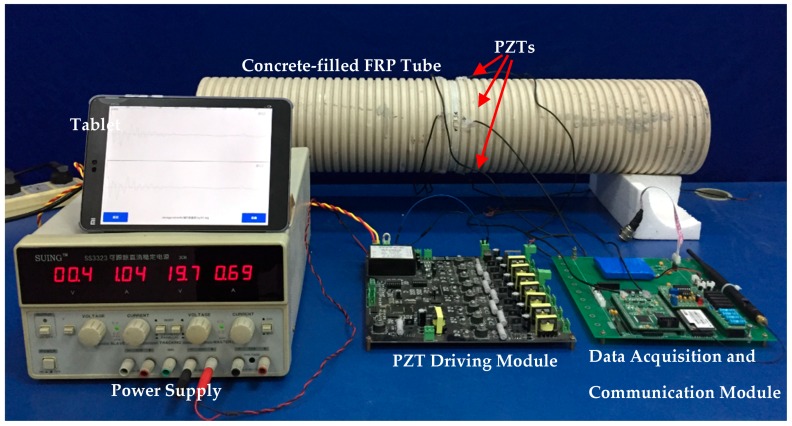
Experimental setup.

The experimental setup of the concrete infill detection of the CFFT using PZT-based TOF is shown in Figure 7. The CFFT was fabricated with four PZT actuators installed 90 degrees apart from each other on the wall of a FRP tube (shown in Figure 1). Four concrete infill conditions were created in sequence according to the analysis of the numerical simulation, including empty, 1/3, 2/3, and full concrete infill conditions. The configurations of empty, 1/3 and 2/3, and full concrete infill conditions were used to simulate the concrete infill process during the concrete casting of the CFFTs, which require continuous monitoring to ensure the proper coupling between the FRP tube and the infilled concrete. For existing CFFTs, voids and debonding may develop due to inadequate compaction and concrete shrinkage, which were also approximated by the 1/3 and 2/3 concrete infill conditions. During the experiments, the PZT driving module actuated one of the PZTs; at the same time, the data acquisition and communication module collected the waveforms from the receivers and sent the data to a tablet via Wi-Fi. Since the data acquisition and communication module has only two input channels, the two PZT receivers next to the PZT transmitter were used to receive the waveforms.

**Figure 8:**
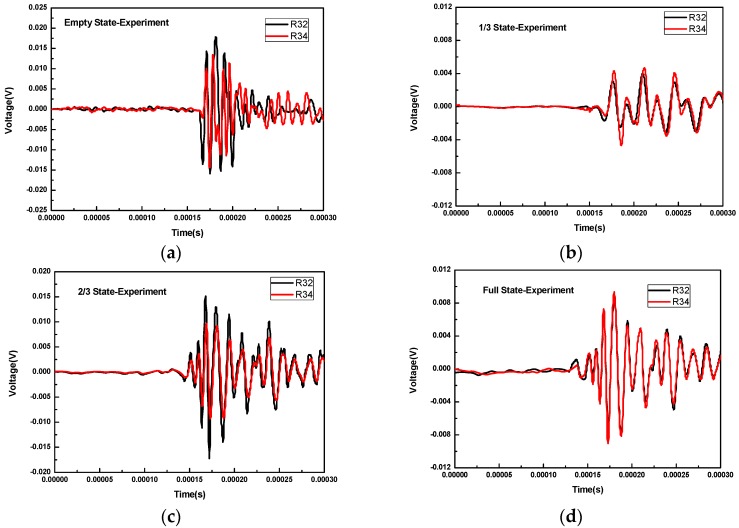
The waveforms received by R2 and R4 for different concrete infill conditions when R3 is used as transmitter: (**a**) the empty concrete infill; (**b**) the 1/3 concrete infill; (**c**) the 2/3 concrete infill; (**d**) the full concrete infill.

Figure 8 shows the waveforms received by R2 and R4 for different concrete infill conditions when R3 was used as transmitter. For all these concrete infill conditions, the waveforms of R2 and R4 almost overlap in that conditions are symmetrical for R2 and R4 when R3 was the excitation source. This overlap is also predicted by the numerical simulation. When there is no concrete infill, as shown in Figure 8a, the wave propagates via the wall of the FRP tube and thus requires more time to reach R2 and R4. In 2/3 and full concrete infill conditions, the wave propagates via the concrete. As can be seen from Figure 8c,d, the arrival time of the head wave is smaller. However, for the 1/3 infill condition, the wave propagation path includes the path in concrete medium and the one along the wall of the FRP tube; thus, the arrival time of the wave should lie between the one for the full condition and the one for the empty condition.

**Figure 9:**
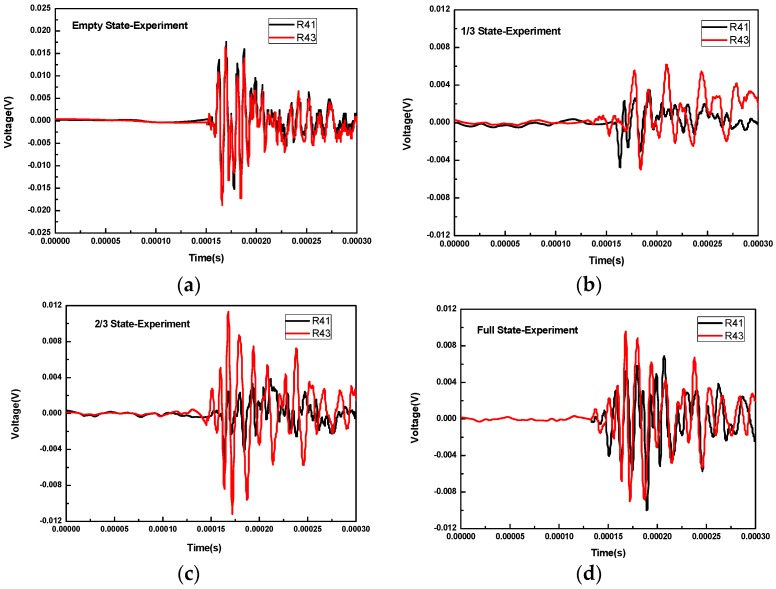
The waveforms received by R1 and R3 for different concrete infill conditions when R4 is used as transmitter: (**a**) the empty concrete infill; (**b**) the 1/3 concrete infill; (**c**) the 2/3 concrete infill; (**d**) the full concrete infill.

Figure 9 shows the waveforms received by R1 and R3 for different concrete infill conditions when R4 was used as a transmitter. In this case, the waveforms of empty and full concrete infill conditions are not much different from those in Figure 8. Attention should therefore be paid to the 1/3 and 2/3 concrete infill conditions. For the case of the 1/3 concrete infill, the path taken by the wave from the R4 transmitter to R1 is via the wall of the FRP tube, while the path to R3 contains the wall of the FRP tube and the concrete. As can be seen in Figure 9b, the head wave of R43 arrives earlier than that of R41. Likewise, for the case of the 2/3 concrete infill, the path taken by the wave from the R4 transmitter to R1 contains the wall of the FRP tube and the concrete, while the path to R3 is merely via the concrete medium. It can be seen in Figure 9c that the head wave of R43 arrives earlier than that of R41.

**Figure 10:**
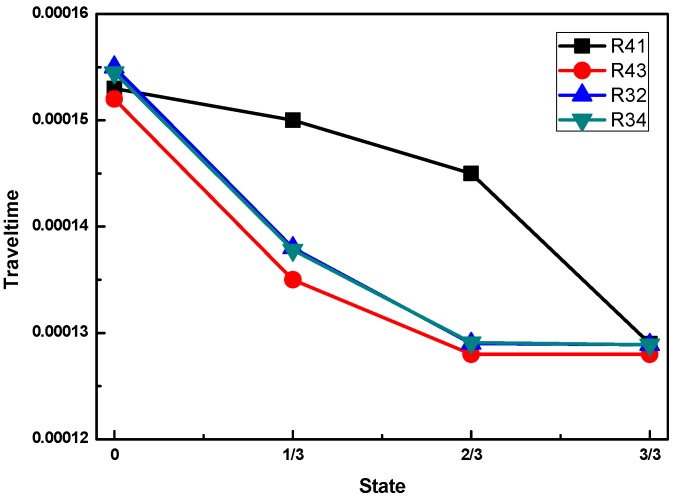
The arrival time of different concrete infill conditions from the experimental study.

In this experimental study, the arrival time of the different receivers for the different concrete infill conditions is shown in Figure 10. Comparing the experimental results with the numerical simulation results in Figure 6, a strikingly similar shape of the wave travel time curve is observed, except that R31 and R42 are not included in the experimental investigation. It is worth noting that the exact values of the travel time are not well-matched between the numerical calculations and the experimental measurements. The main reason for this deviation is that the velocities and material properties adopted in the numerical simulation were assumed based on the manufacturer datasheet. However, the agreement can be improved by using the exact material properties of the adopted materials through measurement. In summary, based on the ultrasonic TOF, the concrete infill conditions of a CFFT can be determined.

## 4. Conclusions

In this paper, the feasibility of using the ultrasonic TOF method to detect the concrete infill condition of CFFTs was examined both numerically and experimentally. By investigating the snapshots of different concrete infill conditions in CFFTs in a numerical simulation, it was found that the energy of an ultrasonic wave is concentrated in the concrete material. The wave propagates faster in concrete than in the FRP material. The arrival time of the wave amplitude of the received waveforms contains critical information about the concrete infill condition inside the FRP tube. In a full concrete infill condition, the wave travels with the velocity in the concrete material, and the time of flight between the transmitter and any receiver is at a minimum value. If a void is created in between the transmitter and any receiver, such as the 1/3 and 2/3 concrete infill conditions, the wave propagation path will be directed to the FRP tube before it reaches the receiver, thus increasing the ultrasonic TOF. The ultrasonic TOF is at a maximum when there is no concrete infill in the CFFT.

These results show that the PZT-based time-of-flight method is able to detect and locate the concrete infill condition of CFFTs. The proposed method can be adopted for new structures for concrete infill monitoring during concrete casting and to existing structures for void detection and determination. This method benefits from an easy operation, high flexibility, a low cost, and remote sensing, and is therefore a promising candidate for the concrete infill monitoring of concrete-filled FRP tubes.

## Figures and Tables

**Table 1:** Material properties for the numerical simulation.

Material	Velocity of Longitudinal Wave (m/s)	Velocity of Transverse Wave (m/s)	Density (kg/m^3^)	Inner Diameter (mm)	Outer Diameter (mm)
FRP Tube	1600	800	1500	146	150
Concrete	3500	1800	2000		146
Air	330		5		
